# Stakeholders’ perspectives on models of care in the emergency department and the introduction of health and social care professional teams: A qualitative analysis using World Cafés and interviews

**DOI:** 10.1111/hex.13033

**Published:** 2020-08-25

**Authors:** Marica Cassarino, Rosie Quinn, Fiona Boland, Marie E. Ward, Rosa McNamara, Margaret O’Connor, Gerard McCarthy, Damien Ryan, Rose Galvin, Katie Robinson

**Affiliations:** ^1^ Faculty of Education and Health Sciences Ageing Research Centre Health Research Institute School of Allied Health University of Limerick Limerick Ireland; ^2^ Emergency Department Our Lady of Lourdes Hospital Drogheda Drogheda Ireland; ^3^ HRB Centre for Primary Care Research Royal College of Surgeons in Ireland Dublin Ireland; ^4^ School of Psychology Trinity College the University of Dublin Dublin 2 Ireland; ^5^ Emergency Department St. Vincent University Hospital Dublin Ireland; ^6^ Department of Ageing and Therapeutics University Hospital Limerick Limerick Ireland; ^7^ Faculty of Education and Health Sciences Graduate Entry Medical School University of Limerick Limerick Ireland; ^8^ Emergency Department Cork University Hospital Cork Ireland; ^9^ Retrieval, Emergency and Disaster Medicine Research and Development Unit (REDSPoT) Emergency Department University Hospital Limerick Limerick Ireland

**Keywords:** emergency department, health and social care professionals, implementation, interdisciplinary care, participatory research, World Café

## Abstract

**Background:**

There is some evidence that health and social care professional (HSCP) teams contribute to enhanced patient and process outcomes in increasingly crowded emergency departments (EDs), but the views of service users and providers on this model of care need investigation to optimize implementation.

**Objective:**

This qualitative study investigated the perspectives of key ED stakeholders about HSCP teams working in the ED.

**Methods:**

Using a participatory design, we conducted World Café focus groups and individual interviews in two Irish hospital sites with 65 participants (purposive sampling) including ED patients and carers/relatives, ED doctors and nurses, HSCPs and pre‐hospital staff. Data were thematically analysed using NVivo software.

**Results:**

Participants reported that ED‐based HSCP teams could improve quality and integration of care and staff experience (Theme 1) and would be appropriate for older adults with complex needs and non‐urgent complaints (Theme 2). Concerns were raised about operational and relational barriers to implementation (Theme 3), and changes in processes and culture were considered necessary for HSCPs to work successfully in the ED (Theme 4). In contrast to service providers, service users’ concerns centred on the importance of positive communication and relations (Theme 5).

**Conclusions:**

Our study indicates potential acceptability of HSCP teams working in the ED, especially to care for older adults; however, operational and relational aspects, particularly developing interdisciplinary and integrated care, need addressing to ensure successful implementation. Differences in priorities between service users and providers (relational vs operational) highlighted the usefulness of gathering views from multiple stakeholders to understand ED processes.

## 1 BACKGROUND

Emergency departments (EDs) worldwide are experiencing increasing numbers of attendances due to population ageing, multimorbidity and limited resources in primary and acute care, which impact negatively on patient flow and outcomes.^1^, ^2^ Calls for ED quality improvement strategies have highlighted the need to identify cost‐effective models of care in terms of optimization of workforce (eg skill mix) or operations (eg fast‐track systems).^3^, ^4^ Encouraging evidence suggests the potential benefits of diversifying the ED workforce in terms of both promoting interdisciplinary work^5^, ^6^ and extending the scope of practice of health and social care professionals (HSCPs), as their specialized skills can enhance decision making and quality of care, particularly when working within a multidisciplinary team.^7^, ^8^ ED‐based HSCP teams are common in some countries, such as Australia,^9^ where they have demonstrated some level of effectiveness in improving patient outcomes^8^; however, this type of service is in its infancy elsewhere, and there is limited evidence of the factors that may influence its implementation.^4^

Introducing new models of care to settings with well‐established organizational structures is a complex process, particularly if involving workforce changes.^10^ The Theoretical Domains Framework of behavioural change^11^ suggests that the implementation of new practice may require modifications at multiple levels, including individuals, organizations, services and systems. Engaging stakeholders who receive or provide health care has become increasingly instrumental to identify factors of implementation based on experiences of care,^12^, ^13^, ^14^, ^15^ as well as understanding pathways to safer and more effective patient care.^16^ Studies of users’ ED experiences suggest that patients value integrated and competent care and empathetic communication.^17^, ^18^, ^19^ Similarly, ED service providers perceive positive communication and interactions as key enablers of health‐care change,^10^, ^20^, ^21^, ^22^ although organizational aspects that may impact their daily operations are also valued, such as receiving appropriate education about new processes and adequacy of resources.^23^

Building on the Medical Research Council's Framework for the development and evaluation of complex interventions^24^ and on the findings of a recent systematic review on HSCP teams in the ED,^8^ the present study aimed to explore the views of various ED stakeholders (service users and providers) on the role and impact of HSCP teams working in the ED, including the perceived value of HSCPs’ extended scope of practice,^25^, ^26^ potential concerns about feasibility and acceptability,^10^, ^26^ and the needs of specific patient populations.^27^ To this end, we employed a World Café focus group format (http://www.theworldcafe.com/), which has been used successfully to investigate mechanisms of change in health‐care settings.^28^, ^29^ This participatory approach promotes the engagement of different stakeholders in an inclusive, equitable environment^30^ where diverse perspectives are encouraged and valued, thus providing rich insights on the complex dynamics and processes that may enable or hinder the introduction of an ED HSCP team.

## 2 METHODS

This qualitative study using a participatory design adhered to the Consolidated Criteria for Reporting Qualitative Research (COREQ)^31^ guidelines. A full checklist is included in File S1.

### 2.1 Participants

Participants for this study (N = 65, age range = 25‐75 years; 76.9% female) included individuals who had visited the ED at one of the two study sites (details below) in the past 12 months either as patients or as carers/relatives, aged ≥18 years, living in the community (not inpatient) and having conversational English language skills; staff members including ED doctors and nurses, HSCPs working in the ED or in the hospital, and pre‐hospital staff (advanced paramedics or emergency medical technicians) in full‐ or part‐time employment in the hospital for at least 12 months (thus, familiar with the ED/hospital procedures).

We recruited participants through purposive sampling, informed by the inclusion criteria described above. Participants’ selection was conducted by research team members (DR and RQ) who acted as gatekeepers at the hospital sites, providing prospective participants with information sheets and organizing in‐hospital advertisement through flyers and screen display. Prospective participants contacted one member of the research team (MC) who provided further details about the study, asked to read and sign the consent form and arranged participation either in one of the focus groups or in an individual interview. Seven out of 72 prospective participants refused to take part. The gatekeepers were blinded to participants’ recruitment and data collection (ie not informed on whether prospective participants agreed or not to take part, and not involved in data collection) to maintain confidentiality and to ensure voluntary participation.

In order to boost recruitment, we also employed snowball sampling and open advertisements through social media and flyers distributed in the local community (eg community centres, GPs, supermarkets). Recruitment occurred iteratively until data saturation was agreed by two authors (MC and KR).^32^

This multisite study adheres to the Declaration of Helsinki and received ethical approval from the Health Service Executive (HSE) Mid‐Western Regional Hospital Research Ethics Committee (reference number: 040/18) and from the HSE North East Area Research Ethics Committee (reference number: 3/9/18).

### 2.2 Settings

Four World Café’ focus groups (n = 53) were held in two separate Irish hospital sites in the Mid‐West and North‐East regions (with similar patient populations). At both sites, the ED did not have a dedicated HSCP team at time of data collection, with HSCP services provided mainly in the ward or on‐call in the ED at the discretion of the medical team. Three focus groups took place in a meeting room on the hospital campus, hosting between 10 and 15 participants each. One focus group took place in a meeting room at a hotel venue near the hospital (n = 13). Participants unable to take part in the focus groups completed individual interviews (n = 12) either over the phone or in person.

### 2.3 World Cafés

The World Café procedure followed in this study is described in File S2. Three members of the research team (KR, MC and RG) facilitated the World Cafés, all experienced in conducting qualitative research. Participants were invited to brainstorm and discuss two questions:

1. What role could HSCPs play in the ED?
2. How would you feel about HSCPs working in the EDs?

While we were interested in several aspects of a HSCP team model of care (implementation process, impact), we opted for broad questions in line with the World Café guidelines to enable participants to generate ideas and views freely. Participants were asked to focus on five HSCP professions: clinical pharmacists (CPs); medical social workers (MSWs); occupational therapists (OTs); physiotherapists (PTs); and speech and language therapists (SLTs). These five disciplines were chosen based on the evidence on extended scope of practice for HSCPs in the ED.^7^, ^8^

At the end of the focus group, participants completed a short demographic survey collecting data on their sex, age and stakeholder type.

### 2.4 Individual interviews

The individual semi‐structured interviews (n = 12; 67% users, 33% providers) explored the same questions investigated in the World Café focus groups with the addition of prompts and follow‐up questions (Interview schedule in File S3); participants completed the demographic survey at the end of the interview. Interviews lasted 20‐40 minutes. No repeat interviews were held.

### 2.5 Data analysis

The participants’ responses in the group and individual interviews were audio‐recorded electronically and transcribed in full; we also took pictures of the paper sheets of each World Café table discussion to include in the transcript; recordings and paper sheets from the World Café’ were matched for each table discussion, checked for consistency and analysed as one document including both transcript and paper sheet picture. Participants did not check transcripts after data collection. While the study questions were developed and agreed by consensus by the research team to ensure relevance to both service users and providers, two female authors (MC and KR) with expertise in the area of health and health service delivery conducted data transcription, analysis, interpretation and write‐up; MC has experience in quantitative and qualitative methods in the area of health; and KR has extensive expertise and experience in qualitative research in the area of health and health service delivery. In keeping with guidelines on reflexivity, the researchers (MC and KR) critically evaluated the impact of their position (personal characteristics, beliefs and relevant experiences; having no professional ED experience and single ED attendances each as service users/relatives of service users) on the research process.^33^ The researchers (MC and KR) strove to avoid leading language during data collection or sharing personal experiences while maintaining an empathetic attitude. The research team reviewed the data analysis to ensure that multiple perspectives were considered.

Transcribed recordings and pictures of paper sheets were entered into the qualitative data analysis software NVivo 11 Pro (QSR International Pty Ltd). Data saturation was determined by two researchers (MC and KR).^32^ A descriptive inductive approach to data analysis was employed to provide a rich and detailed account of the data, adhering to the six‐stage guide to thematic analysis described by Braun and Clark^34^, ^35^: (a) familiarization with the data through repeated reading; (b) identification of initial codes; (c) sorting of codes into potential themes; (d) review and discussion of themes by the two researchers (MC and KR) to ensure internal homogeneity and external heterogeneity; (e) naming and definition of themes; and (f) final analysis and generation of underlying story linking the themes. The Results section includes anonymized extracts of transcripts to support the identified themes, with an indication of the stakeholder type (user or provider) and whether quotes come from interviews (IV) or World Cafés (WC). Both authors selected quotes that exemplified each theme and then agreed on the quotes to include by consensus.

## 3 RESULTS

The characteristics of our sample are provided in File S4, with details about participant distribution across focus groups and interviews. We identified five themes related to the two study questions, described hereafter.

### 3.1 HSCP teams working in the ED could improve quality and integration of care and staff experience

Overall, both service users and providers expressed positive views about HSCPs working in the ED, with benefits highlighted for patients, staff and the hospital more broadly:

I think it would be a massive benefit to everybody’s experience for there to be an Allied Health team down here (User‐IV2)

Considering benefits for patients, both users and providers described having the HSCP team in the ED rather than the ward as contributing to timely assessment/treatment (eg reduced ED length of stay) thanks to their specialized skills. Providers also highlighted the potential for improved quality and safety of care and a more integrated service through multidisciplinary decision making and better linkages with community services, leading to safer discharges home and reduced risk of secondary conditions:

I think that having a comprehensive multidisciplinary team in the emergency department is very beneficial for the patient and very important and I do think that it can help to provide a better clinical service for patients (Provider‐IV6)

From a user's perspective, timely and multidisciplinary care was associated with increased satisfaction, reduced carer's anxiety and, to a lesser extent, improved patient education (eg advice on available services in primary care) and fewer ED returns.

Staff members saw a HSCP team as promoting lighter workload for ED medical staff as well as hospital staff who could otherwise be on call in the ED; however, this benefit would be really felt if the HSCPs were to work out of hours:

A lightened workload because if, say that hypothetically there was access seven days a week 24/7 to the healthcare professionals, I would come in thinking, like, ‘Well, this patient needs a brace put on’ and it’s going to get done quicker so then you don’t have to go bleeping or ringing somebody, so you are spending less time going around running after these kind of assessment teams (Provider3‐WC3)

Another benefit for staff was shared decision making and enhanced multidisciplinary team working, thus the ability to integrate different perspectives in order to make effective decisions:

The collective brain on everybody. So, putting them all together (Provider1‐WC3)

Staff member also described interdisciplinary teamwork as an opportunity for upskilling and for the promotion of networking/support and job satisfaction.

Lastly, service providers mentioned the potential cost‐effectiveness of having specialized workforce in the ED:

I think that ultimately it would cost the HSE [Health Service Executive] less, because the time they would stay in hospital is shorter (Provider2‐WC2)

### 3.2 HSCP teams have a role in the ED mainly for non‐urgent complaints

**Figure 1:**
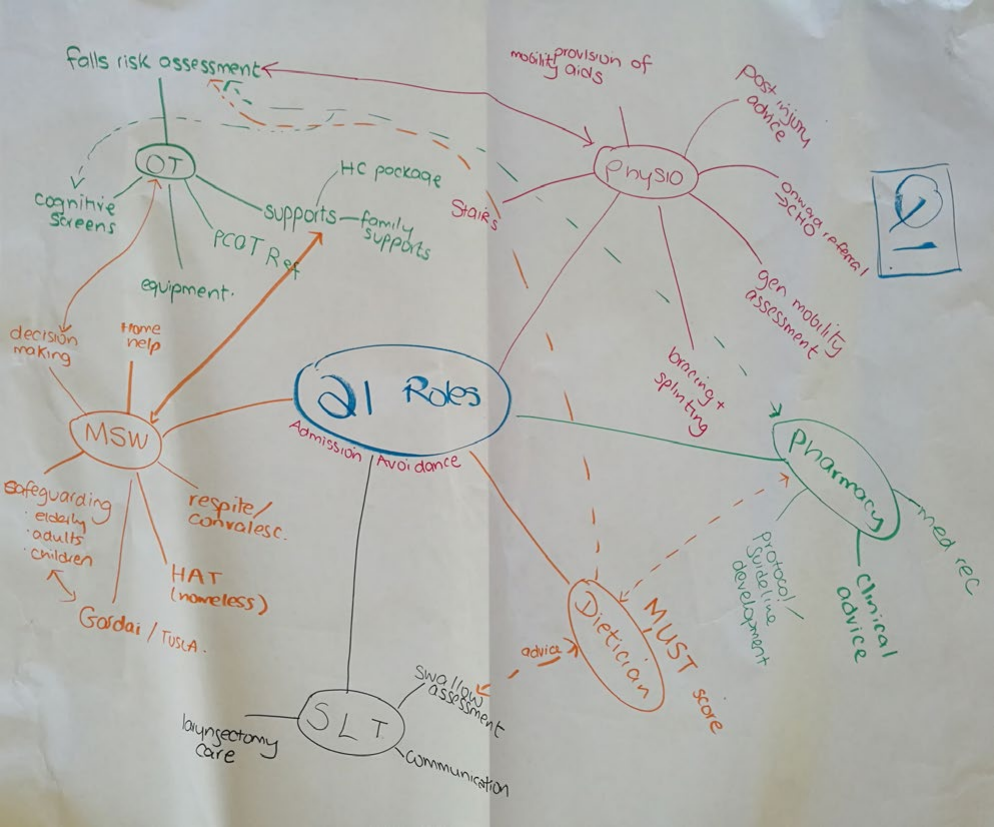
HSCPs’ competencies working as a multidisciplinary team and alone. (Source: WC1). Gardai, An Garda Síochána, the Irish Police Service; HAT, Homeless Action Team; HC, home care; Med Rec, medication reconciliation; MSW, medical social worker; MUST score, Malnutrition Universal Screening Tool score; OT, occupational therapist; PCOT Ref, primary care occupational therapist referral; Q1, question 1; SLT, speech and language therapist; Tusla, Irish Child and Family Service

Given the focus of this study on team care, both users and providers discussed how different HSCPs could collaborate; an example of this discussion is illustrated in Figure 1.

Users and providers agreed on the relevance of an interdisciplinary HSCP team for patients with non‐urgent complaints but complex demands, especially older patients with frailty issues or fall risk:

Physiotherapists and Occupational Therapists would be very important to assess an older patient who has experienced a fall (User‐IV4)

HSCPs working as team were perceived as increasing patient safety in a holistic manner; a HSCP team could for instance provide mobility assessment, treatment and education (PT and OT), together with safe discharges through linkage with community services (MSW, OT, PT and CP), particularly for older patients and frequent users. Other index complaints that could be addressed by the team included the management of chronic disease and treatment of acute injuries.

While considering HSCPs in teams, participants engaged in an in‐depth discussion about the competencies of specific HSCPs. OTs were designated as providing functional assessment and appraising the need for aids and equipment, particularly for older frail patients. PTs were described as crucial to optimize care for managing musculoskeletal conditions and orthopaedics. CPs were frequently associated with enhanced medication management and education. SLTs were seen as crucial for communication and swallow assessment/management, especially for stroke patients. Most participants agreed that MSWs were instrumental to safeguard vulnerable people, particularly older individuals and children. Some participants discussed also the role of other HSCPs, particularly dieticians, who have specialized skills to provide dietary and weight assessment. Both service users and providers described the roles of MSWs, SLTs and PTs, whereas inputs on OTs and CPs were provided by staff members only, and some service users were not aware of what their role might be in the ED.

### 3.3 Concerns about operational and relational challenges for HSCPs working in the ED

When asked how they felt about HSCPs working in the ED, concerns were expressed alongside positive expectations. Participants discussed potential barriers to successful implementation in terms of the ED physical space, operational and relational aspects, the community and the broader health‐care system.

Considering the physical space, both service users and providers felt that limited space and crowding in the ED would limit the ability of HSCPs to conduct an assessment efficiently and maintain the patient's privacy:

You are not going to be able to spend a half an hour or an hour with a patient. You might need to see them for ten minutes and basically assess them within their tiny ED cubicle and make a decision on their function, their cognition, their consciousness, and decide ‘Well, they should be safe enough to go home’ (Provider‐IV5)

At an operational level, HSCPs’ absence from the ED outside business hours (ie night, weekend) was seen by both users and providers as potentially leading to a two‐tier care service for patients, as well as increasing pressure on existing ED staff who work out of hours. Furthermore, both service users and providers felt that the current ED system whereby the onus of decision making is overwhelmingly on doctors limits the HSCPs’ role. Providers also raised concerns that adding extra staff members could cause a burden on the system, both in terms of more individuals being physically present in limited space and in relation to more difficult decision making given the high volumes of attendees with complex demands. Some service users felt that HSCPs could compound communication challenges in the ED; thus, adding more professionals would need to be carefully planned:

My worry is that if you are going to introduce lots of different professionals into a situation that is already pretty fragmented, what supports are you going to put in place for these people to actually communicate, or what spaces are you going to create to ensure that this adds something rather than … And currently, if you put a physio or an SLT into our experience, it would be just another person in a long chain of breakdowns of communication (User‐IV1)

Operational issues were closely linked to challenges at the community level. Some providers expressed concerns about potential growing patient demands on the ED and crowding linked to increased awareness of HSCP services in the ED, coupled with limited access to community services. Also, having an ED‐based HSCP team without improving community services could limit opportunities to discharge patients safely:

Increased demand on service with limited community resources (Provider5‐WC1)

Some participants, particularly users, felt that limited awareness of HSCP roles may impact negatively on acceptability and the HSCPs’ capacity to build positive relationships in the ED. Some users and providers raised issues with HSCPs being the first point of contact in the ED and some questioned the HSCPs’ ability to diagnose rather than just treat:

I have great time for physios and a respect for what they do and certainly they know how to deal with the problem, but it’s not a physio’s job either. I mean, they don’t look at scans. They don’t have that so it’s not their job either to know what exactly is wrong, you know. They just treat the issue (User‐IV9)

Several staff members identified potential ‘resistance to change’ (Provider6‐WC1) from existing ED staff, and the ED busyness and staff volumes as important relational barriers:

As far as I know, there is like 150 nurses here all scheduled at different times and whatever but yeah, I mean, it’s hard to get to know people in terms of the team‐building element. The staff turnover is huge (Provider‐IV3)

Lastly, system barriers included mainly limited financial resources in place to recruit and retain new staff in the ED, but also concerns about cost‐effectiveness for the hospital (ie Is funding for HSCPs better spent on the ward or in the ED?) and that perhaps there might be more important issues to tackle before adding HSCP services, as, for example, increasing bed availability:

In a perfect world, it would be wonderful, but I don’t think it’s a realistic achievable thing to be honest. I think, to be honest, the general public will be a lot happier with less people in corridors and that the people who are in the corridors are cared for properly. Then, after that then, once you have that sorted out, then sort everything else out. I think you are jumping ahead a little bit (User‐IV2)

### 3.4 Changes in processes and culture needed for HSCPs to work successfully in the ED

The concerns raised in theme three were discussed in the context of changes in processes and culture needed for HSCPs to work successfully in the ED; these included strategies to positively integrate the HSCP team within the ED team, such as promoting acceptability through staff education and defining the appropriate competencies for the team:

We were talking to the girls [colleagues] here about, say, how it would actually work within ED so having, the whiteboard that they have down there and utilising that to see who actually needs to be referred to us and who needs to be seen and getting them involved and to work alongside with the two ED consultants that are on call on that particular day to see how we are actually managing from a multidisciplinary perspective (Provider3‐WC1)

Promoting acceptability and trust was felt as a crucial enabler of change by both users and providers:

In terms of acceptability, trust is obviously a big thing, in terms of what the roles of the person are, what their remit is, the acceptability to other staff in the ED, and obviously the patients. So are patients happy to be seen by a physio instead of a traditional doctor or nurse (Provider4‐WC2)

Considering implementation, service providers recommended involving HSCPs at senior level, given the complexities of ED team care, and liaising with organizations that have already implemented this model of care. To change ED culture and established practices, participants suggested education about the benefits of HSCPs’ specialist skills and potential outcomes alongside clarification of role boundaries:

I think communication and education is going to be huge in terms of setting it up, you know? It’s not a … You know, there will have to be a very clear remit in terms of what their role is and how it’s going to play out and what the objectives are (User‐IV3)

HSCPs need to work in new ways in the ED and be comfortable with working outside the traditional scope of practice; this was identified by some as requiring a culture change as all ED team members needed to share a common goal:

So, they’re part of the one team, but everybody has to have the same focus or goal in that it’s an emergency department. You deal with the emergencies and then you refer on to the community for the non‐emergencies, so I think it’s a cultural and a way of thinking but it’s not traditional – it’s about getting people home but getting them home safely (Provider‐IV5)

### 3.5 Users’ concerns about their ED experience centring on communication and feeling that they are in good hands

Participants also provided suggestions on issues that were not necessarily related to the HSCP model of care but would improve the ED experience; many suggestions related to the need for improved staff/patient communication, staffing resources and the quality of ED non‐clinical services.

Good communication was felt as key to improve ED care by both service users and providers: service users discussed more staff/patient relationships, whereas service providers highlighted the need for improved communication between staff members. Some users reported negative relational experiences of care and poor communication by service providers in the ED. Some service users described the ED as a ‘de‐humanising’ place where patients are treated as ‘conditions rather than individuals’ (User‐IV1). To this end, service users suggested that implementing models of ED care that promote positive staff/patient communication and active patient involvement in the care process would enhance patient satisfaction and trust. The desire to feel as being in ‘good hands’ re‐occurred across users’ accounts of both positive and negative past ED experiences:

Having someone who was specialised was very reassuring to begin with and then somebody who was thorough and also who explained what to look out for, even really briefly, it just meant that we felt reassured going home that we would know what to do and when to worry and when not to, you know? (User‐IV11)

In terms of staffing resources, it was felt that ED doctors should be better staffed and that professionals providing personal care would reduce the current heavy workload on ED nurses as well as promote patient dignity:

I really feel strongly that there needs to be two carers at all times on the corridor, helping elderly people and changing them and giving them a little bit of dignity (User‐IV2)

Other suggestions included improving hot food services for patients on trollies on the corridor, providing refreshments for carers, improving pre‐hospital resources (ie number of ambulances) and introducing ED waiting time monitors.

## 4 DISCUSSION

### 4.1 Summary of findings

This study used a participatory approach to understand the views of a variety of stakeholders on introducing team‐based HSCP services in the ED. Overall, HSCPs working in the ED were seen as having the potential to enhance the quality, timeliness and safety of care and promote integrated care for patients, facilitate lighter workload for existing staff members and potentially lower costs for the hospital. Participants agreed that ED patients with non‐urgent complaints and with complex needs, particularly frail older adults, would benefit the most from having a HSCP team. Despite identifying multiple benefits, concerns were raised about barriers to feasibility, including limited ED physical space, working hours, care protocols, communication and acceptability. Given the novelty of ED‐based HSCP services in the Irish context, our findings have policy and practice implications in that changes in the scope of ED practice and a more interdisciplinary culture of care would be necessary for HSCPs to work successfully in the ED. Service users in particular highlighted the need to improve relational staff/patient dynamics in the ED and the quality of non‐clinical services. Borrowing from one participant, an appropriate summary of stakeholders’ views is that the introduction of ED‐based HSCP teams is ‘positive with challenges’.

Notably, in our analysis we found that service users focused mainly on relational aspects, whereas service providers discussed more operational issues. One reason for this diversity may lie in the fact that service users appeared to be less aware of the operational aspects of an ED as well as the competencies of HSCPs. Furthermore, users brought up issues with their ED experience that might not necessarily apply to HSCPs specifically, but which they felt would have a significant impact on how future patients might accept having more professionals in the ED.

### 4.2 Results in the context of the current literature

In line with a systematic review of ED stakeholders’ perceptions of HSCPs,^36^ our study showed overall acceptability both among service users and providers, with similar benefits reported in terms of timely, safe and integrated care, particularly for older patients with complex needs. In line with our findings, qualitative studies in Australia and Sweden have suggested comprehensive, integrated and interdisciplinary care as key to promote better health‐care services across acute and primary settings, especially for older patients.^5^, ^29^

On the other hand, in line with the Theoretical Domains Framework,^11^ participants raised concerns about potential operational and relational influences on implementation: feasibility issues related to space, time resources and cost‐effectiveness, as well as challenges in optimizing teamwork and care pathways, were consistent with the international literature.^18^, ^21^, ^36^, ^37^ Also in line with previous studies,^21^, ^36^ patients and staff feared the negative impact of limited working hours on quality and continuity of care; hospital HSCP services in Ireland are conventionally within business hours, but this may not fit well with the 24/7 ED opening hours. These issues should be carefully considered both at policy and practice levels to ensure successful changes in models of emergency care.

The discussed need to optimize care protocols and team communication for the HSCP team to enhance quality of care resonate with findings of previous studies suggesting the need for improved communication, teamwork and operations when caring for patients with complex needs, especially if older.^22^, ^38^ Importantly, our finding that ED service users and providers attached different values to relational and operational aspects, which aligns with the extant literature,^18^, ^20^, ^39^ shows that individuals’ perspectives depend strongly on their specific roles and experiences, thus supporting the importance of engaging multiple stakeholders in order to understand factors of implementation.

### 4.3 Strengths and limitations

While in keeping with previous literature, our study is, to the best of our knowledge, the first to investigate views on HSCP teams working in the ED through the engagement of multiple stakeholders. Employing a World Café format enabled the creation of an inclusive space for inquiry where different stakeholders had an opportunity to sit together and discuss issues relevant to them. On the other hand, individual interviews provided an additional space for participants who were unable or unwilling to be in the focus groups; the interviews offered deep insights about individuals’ experiences in the ED, thus providing a richer understanding of the reasons behind the perspectives that had emerged during the focus groups.

Our study is not without limitations. Although we collected data in two separate sites in Ireland, generalizability of findings might be limited given the specific contextual characteristics of health‐care systems in different countries; nonetheless, our results are in line with those of the existing international literature.^18^, ^21^, ^36^ Despite its many benefits, the World Café format has some disadvantages: Firstly, as for other qualitative investigations, social desirability may have influenced the participants’ contributions. Furthermore, the World Café notes were more difficult to contextualize and interpret than audio‐recorded data, although we matched notes and transcripts where possible. As the World Café table membership rotated, we were not always able to identify specific respondents in the recordings; also, although all the five relevant HSCP disciplines were represented in our sample, we did not record how many participants from each HSCP discipline were present. While we involved different stakeholders, our sample did not include policy‐makers or members of the hospital Executive, who could have contributed further insights on factors of implementation; lastly, more service users participated in interviews than focus groups; thus, it is possible that group discussions could have been different if more users were present.

## 5 CONCLUSIONS

In this qualitative participatory study, ED service users and providers highlighted potential benefits of having HSCP teams working in the ED, particularly for enhancing the care of patients with complex needs; however, several factors including communication, acceptability, physical space, interdisciplinary work and community resources arose as issues to be carefully considered in order to optimize the implementation of this model of care. The study has practical implications for the implementation of HSCP teams in EDs and for future research on ED‐based interdisciplinary work, which have both been suggested as potential cost‐effective strategies to improve ED care.

## CONFLICT OF INTEREST

The authors declare that they have no competing interests.

## AUTHORS' CONTRIBUTIONS

MC and KR were major contributors in writing the manuscript. MC, KR and RG designed the study. MC, RG and KR conducted data collection and analysis. RQ, DR, FB, MEW, RMN, MOC and GMC participated in the project design and critically appraised and edited the manuscript. RG is the guarantor of the study. All authors read and approved the final manuscript.

## Data Availability

Due to confidentiality and the nature of the consent obtained, the interview transcripts cannot be shared. For further information related to this data set, please contact the corresponding author.
