# Supplementary material for: Stakeholders’ perspectives on models of care in the emergency department and the introduction of health and social care professional teams: A qualitative analysis using World Cafés and interviews
Source: Health Expect. 2020 Aug 25;23(5):1065–73. doi: 10.1111/hex.13033 (PMC7696138; doi:10.1111/hex.13033)
Supplement: Supplementary file 2 [file HEX-23-1065-s002.docx]

**Title**: Stakeholders’ perspectives on models of care in the emergency department and the introduction of health and social care professional teams: A qualitative analysis using a World Café methodology

**Supporting Information File 2 – World Café procedure**

The World Café principles and guidelines emphasise the importance of creating a hospitable environment (i.e. a café-style ambience) where individual and collective knowledge and ideas can be shared. At each site, the venue of the meeting was set with four to five small round tables covered with self-adhesive paper-sheets. Each table had coloured water-based markers, pens, and label stickers to encourage participants to note their ideas on the paper-sheet. Flowers, candle-lights and mellow music were used to create a welcoming and warm environment, and participants were offered light refreshments.

The facilitators set the context to the event by describing the aim of the meeting and providing a clear definition of HSCP figures of interest. The World Café principles and ground rules for participation were discussed.

Each question was discussed for 15-20 minutes, with participants encouraged to self-facilitate their own discussions. A scribe identified from within the group recorded discussions onto the paper-sheet. Participants rotated around tables in between the two questions, while the scribes remained at the same table to provide a brief summary to the incoming group about the previous group’s discussion and to encourage cross-fertilisation of ideas and knowledge. After both questions were discussed at each table, the facilitators and scribes summarised the discussion under representative concepts for 10-15 minutes using a visual summary on a flipchart. All participants had the option of commenting on the visual summary.

Further information on the World Café principles can be found at <http://www.theworldcafe.com/key-concepts-resources/world-cafe-method/>
