# Supplementary material for: Stakeholders’ perspectives on models of care in the emergency department and the introduction of health and social care professional teams: A qualitative analysis using World Cafés and interviews
Source: Health Expect. 2020 Aug 25;23(5):1065–73. doi: 10.1111/hex.13033 (PMC7696138; doi:10.1111/hex.13033)
Supplement: Supplementary file 3 [file HEX-23-1065-s003.docx]

**Title**: Stakeholders’ perspectives on models of care in the emergency department and the introduction of health and social care professional teams: A qualitative analysis using a World Café methodology

**Supporting Information File 3 – Interview schedule**

For service users

Can you briefly describe your experience of using the ED at UHL?

Based on your experience, what would you say are important factors for good patient care in the ED?

When visiting the ED, do you recall being seen by a Health and Social Care Professional such as a physiotherapist, occupational therapist, social worker, pharmacist or speech and language therapist?

- If answer no, ask if familiar with HSCPs in the ED (if not, explain)
- If answer yes, ask to briefly describe the types of services provided by the HSCP(s)/

What role do you think Health and Social Care Professionals play in the Emergency Department?

How would/do you feel about HSCPs working in the Emergency Department?

For service providers

Can you describe you experience of working in the ED at UHL?

Based on your experience, what would you say are important factors for good patient care in the ED?

Could you tell me about the frequency with which you interact with HSCPs in the ED and what types of tasks you undertake together? With HSCP I intend any of physiotherapist, occupational therapist, social worker, pharmacist or speech and language therapist.

What role do you think Health and Social Care Professionals play in the Emergency Department?

How would/do you feel about HSCPs working in the Emergency Department?

**For HSCPs**

Can you describe you experience of working in the ED at UHL?

Based on your experience, what would you say are important factors for good patient care in the ED?

What role do you think Health and Social Care Professionals (HSCPs) play in the Emergency Department?

How would/do you feel about HSCPs working in the Emergency Department?
