# Supplementary material for: Stakeholders’ perspectives on models of care in the emergency department and the introduction of health and social care professional teams: A qualitative analysis using World Cafés and interviews
Source: Health Expect. 2020 Aug 25;23(5):1065–73. doi: 10.1111/hex.13033 (PMC7696138; doi:10.1111/hex.13033)
Supplement: Supplementary file 4 [file HEX-23-1065-s004.docx]

**Title**: Stakeholders’ perspectives on models of care in the emergency department and the introduction of health and social care professional teams: A qualitative analysis using a World Café methodology

**Supporting Information File 4 – Participants’ characteristics**

In our sample, stakeholders were represented as follows: ED patients and carers/relatives of patients (16.9%); ED doctors (13.8%); ED nurses (24.6%); HSCP staff members and managers (38.5%); Pre-hospital or clerical staff (6.2%). ED patients and carers reported one-to-four visits to the ED in the 12 months before data collection. Considering staff members, 36.6% had been employed in the hospital for over 10 years, 9.7% between five and 10 years, and 53.6% one-five years.

The participants’ distribution across World Cafés is presented in Table 1

| Table 1 - Participants’ distribution across World Cafés | | | | | | | |
| --- | --- | --- | --- | --- | --- | --- | --- |
|  | Stakeholder type | | | | | | |
|  | Patient | Caregiver | ED doctor | ED nurse | HSCP (including 2 managers) | Pre-hospital | Other (clerical) |
| World Café 1 | 0 | 0 | 6 | 5 | 0 | 0 | 0 |
| World Café 2 | 1 | 1 | 1 | 0 | 10 | 1 | 0 |
| World Café 3 | 0 | 0 | 2 | 5 | 5 | 0 | 1 |
| World Café 4 | 1 | 0 | 0 | 6 | 7 | 1 | 0 |
| **Total** | **2** | **1** | **9** | **16** | **22** | **2** | **1** |

The participants’ distribution across interviews was as follows:

- 3 patients
- 5 caregivers
- 3 HSCPs
- 1 Pre-hospital staff member

Number of interview participants who reported having met HSCPs in the ED or worked as HSCPs in the ED:

- Patients: 2/3
- Caregivers: 2/5
- HSCPs (worked in ED): 2/3
- Pre-hospital: 1/1
